# Warming will affect phytoplankton differently: evidence through a mechanistic approach

**DOI:** 10.1098/rspb.2011.0160

**Published:** 2011-04-20

**Authors:** I. Emma Huertas, Mónica Rouco, Victoria López-Rodas, Eduardo Costas

**Affiliations:** 1 Instituto de Ciencias Marinas de Andalucía (CSIC), Polígono Río San Pedro s/n 11519, Puerto Real, Cádiz, Spain; 2 Departamento de Genética, Facultad de Veterinaria, Universidad Complutense de Madrid, Avenida Puerta de Hierro s/n, 28040 Madrid, Spain

**Keywords:** phytoplankton, climate change, warming, genetic adaptation, ratchet technique

## Abstract

Although the consequences of global warming in aquatic ecosystems are only beginning to be revealed, a key to forecasting the impact on aquatic communities is an understanding of individual species' vulnerability to increased temperature. Despite their microscopic size, phytoplankton support about half of the global primary production, drive essential biogeochemical cycles and represent the basis of the aquatic food web. At present, it is known that phytoplankton are important targets and, consequently, harbingers of climate change in aquatic systems. Therefore, investigating the capacity of phytoplankton to adapt to the predicted warming has become a relevant issue. However, considering the polyphyletic complexity of the phytoplankton community, different responses to increased temperature are expected. We experimentally tested the effects of warming on 12 species of phytoplankton isolated from a variety of environments by using a mechanistic approach able to assess evolutionary adaptation (the so-called ratchet technique). We found different degrees of tolerance to temperature rises and an interspecific capacity for genetic adaptation. The thermal resistance level reached by each species is discussed in relation to their respective original habitats. Our study additionally provides evidence on the most resistant phytoplankton groups in a future warming scenario.

## 1. Introduction

Climate change is now firmly established as a scientific reality, with a variety of emergent challenges for the Earth system in the coming decades. The oceans play a relevant role in modulating the climate system through storage and transport of heat [1], and through the uptake and sequestration of carbon dioxide [2]. According to the Fourth Assessment Report of the Intergovernmental Panel on Climate Change [3], the global mean surface air temperature rose by 0.74°C over the last century, while the global mean sea surface temperature increased by 0.67°C in the same period [4]. As the release of excess CO_2_ to the atmosphere will continue, the planet and some critical ocean regions may soon be warmer than at any time in the past million years [5,6]. It has been predicted that by the end of the 21st century, the sea surface might experience a temperature augmentation between 1.1°C (low CO_2_ emission scenario B1) and 6.4°C (high CO_2_ emission scenario A1FI) [7]. Warming will also be experienced by large freshwater bodies, with a rise of 1–7°C in surface water temperatures being predicted under a forecasted doubling of atmospheric CO_2_ concentrations [8]. Therefore, there is currently a clear research need to understand the effects of warming on aquatic systems.

The anthropogenically driven temperature rise will disrupt the aquatic environment in many ways. Physical changes are expected, such as modifications in circulation and stratification patterns, which will indirectly have drastic results on biogeochemical cycles of essential elements and biota distribution [9–11]. These alterations will ultimately lead to shifts in food web structure and productivity [12,13]. However, owing to the sensitivity of biological processes to temperature, direct thermal effects on aquatic life forms are also anticipated. From a broad perspective, there are three main response options for organisms facing warming: (i) species may disperse to more hospitable habitats, (ii) phenotypic and physiological plasticity may allow species to tolerate the new conditions, or (iii) species may adapt to the new conditions through genetic change via the process of evolution [14]. In particular, drifting life forms whose spatial distribution is primarily determined by the motion of the water column, such as those integrating the plankton community, rely on the two last mechanisms to cope with the increased temperature, considering the environmental selection forcing. Among this diverse group of organisms, phytoplankton (which are central to biogeochemical and ecological services and play key roles in both regulation of atmospheric CO_2_ through photosynthesis and in the maintenance of upper trophic levels) have already been observed to respond to warming. Thus, marine phytoplankton biomass and productivity have been shown to decrease in response to warmer sea surface temperatures, although this diminution has been attributed to the indirect effect of the temperature-driven stratification on the isolation of surface waters from cool, nutrient-rich deeper water [9]. The predicted reduction in nutrient supply to the euphotic layer as a result of increased thermal stratification has been also indicated as a potential mechanism altering phytoplankton community composition [10]. A direct effect of warmer temperature on phytoplankton populations has been also described, as a significant increase in the proportion of small-sized species under higher thermal conditions has been evidenced in both freshwater ecosystems [15,16] and in the marine domain [13,16]. In fact, a gradual shift towards smaller primary producers in a warmer ocean has been foreseen, as temperature has been regarded as the main environmental parameter controlling size distribution in phytoplankton assemblages [17]. Therefore, studies addressing the straight effect of temperature on phytoplankton populations in the context of global warming focus mainly on allometric relationships and seldom have they specifically analysed the capacity of individual phytoplankton cells to efficiently adapt to the increased thermal conditions. At present, therefore, it is unclear whether the shifts in phytoplankton species composition can be attributed to a direct metabolic response to changes in temperature or are an indirect effect of variations in light, nutrients and other abiotic or biotic factors associated with modifications in water circulation and climate [18].

Additionally, the polyphyletic complexity of the phytoplankton community does not allow one to establish a general conclusion about the cell mechanisms conferring tolerance to warming, but undoubtedly genetic adaptation will ultimately determine species success and survival in a new thermal scenario. Here, various common phytoplankton species from a number of major groups were investigated in relation to their capacity to cope with a temperature forcing. We analysed, at individual level, the maximum capacity of adaptation to a gradual warming process in species belonging to distinct ecological niches, and discussed their responses in relation to their respective natural habitats. Additionally, this study provides experimental evidence for assessing how phytoplankters might respond and evolve to the envisaged higher temperatures in the near future.

## 2. Material and methods

### (a) Organisms and growth conditions

Sixteen strains of 12 phytoplankton species obtained from the Algal Culture Collection of the Universidad Complutense (Madrid, Spain) and belonging to four different major phytoplankton groups were examined. Thus, phytoplankters isolated from continental freshwater bodies, coastal marine waters, open ocean waters and symbiotic of corals were used. The exact isolation sites along with the natural thermal variation range during the year are indicated in table 1.

**Table 1.** Isolation sites of the strains subjected to the ratchet experiment and annual temperature range in their natural environments.

isolation site	location (lat/long)	species/strain	cell volume (µm^3^)	isolation temperature (°C)	annual temperature range (°C)
continental water bodies	037° 006′ N; 006° 028′ W	*Scenedesmus intermedius*	207	21	11–29
	037° 003′ N; 003° 22′ W	*Dictyosphaerium chlorelloides*	78	12	5–27
	037° 005′ N; 006° 029′ W	*Microcystis aeruginosa* (*Ma3D*)	117	23	14–31
	037° 005′ N; 006° 029′ W	*Microcystis aeruginosa* (*Ma6D*)	96	23	14–31
	037° 005′ N; 006° 029′ W	*Microcystis aeruginosa* (*Ma7D*)	70	23	14–31
open ocean	032° 000′ N; 062° 000′ W	*Emiliania huxleyi*^a^ (*CCMP 371*)	180	unknown	19–26
	032° 000′ N; 062° 000′ W	*Emiliania huxleyi*^a^ (*CCMP 372*)	48	unknown	19–26
	043° 041′ N; 011° 013′ W	*Isochrysis galbana*	30	14	13–19
	043° 007′ N; 010° 046′ W	*Monochrysis lutheri*	70	15	13–19
coastal waters	038° 059′ N; 008° 022′ E	*Tetraselmis suecica*	357	22	13–25
	043° 023′ N; 008° 023′ E	*Phaeodactylum tricornutum*	122	14	13–19
	036° 007′ N; 006° 023′ W	*Prorocentrum triestinum*	670	21	15–24
	036° 007′ N; 006° 001′ W	*Nitzschia closterium*	16	21	15–24
	036° 007′ N; 006° 001′ W	*Navicula* sp.	102	21	15–24
corals	023° 008′ S; 152° 000′ E	*Symbiodinium* sp. (*CCMP 2429*)	1153	unknown	22–28
	023° 008′ S; 152° 000′ E	*Symbiodinium* sp. (*CCMP 2433*)	1022	unknown	22–28

^a^Isolated at 40 m depth.

The first group, corresponding to phytoplankton from continental waterbodies, comprised one strain of the chlorophyte *Dictyosphaerium chlorelloides* (Naumann) Komárek and Perman, one strain of the chlorophyte *Scenedesmus intermedius* Chodat and three strains (*Ma3D*, *Ma6D* and *Ma7D*) of the cyanobacterium *Microcystis aeruginosa* (Kützing) Lemmermann. *Dictyosphaerium chlorelloides* was isolated from a mountain lake from Sierra Nevada (southwest Spain), whereas the rest of organisms were isolated from a pristine lagoon in Doñana National Park (southwest Spain). Phytoplankton from coastal marine waters comprised: the prasinophyte *Tetraselmis suecica* (Kylin) Butcher, isolated from coastal waters of Sardinia (Italy); the free-living dinoflagellate *Prorocentrum triestinum* Schiller from the continental shelf of the gulf of Cadiz (Spain); and the three diatoms (Bacillariophyceae) *Nitzschia closterium* (Ehrenberg) Smith, *Navicula* sp. and *Phaeodactylum tricornutum* Bohlin, which were isolated from coastal waters of Galicia (Spain). Phytoplankton from oceanic waters comprised marine haptophytes—specifically, two strains (*CCMP 371* and *CCMP 372* from the UTEX stock) of *Emiliania huxleyi* (Lohm.) Hay & Mohler originally obtained from the Sargasso Sea—and *Isochrysis galbana* Parke and *Monochrysis lutheri* (Droop) Green, both isolated from the north central Atlantic. Finally, two strains (*CCMP 2433* and *CCMP 2429*) of *Symbiodinium* sp. (Dinophyceae), extracted from corals of the Coral Sea (south Pacific), formed the group of symbiotic phytoplankton.

For each strain, cultures were re-cloned before experiments by separating a single cell in order to avoid the inclusion of genetic variability that might have occurred in the culture by mutations prior to experiments. After isolation, a single cell was asexually grown until around 500 cells were obtained, which were used to create triplicate bottles of 100 cells. Triplicates were grown axenically during 30 days prior to the experiments in ventilated cell-culture flasks covered with a filter cap (Greiner, Bio-One Inc., Longwood, NJ, USA) containing either 20 ml of BG11 medium (Sigma, Aldrich Chemie, Taufkirchen, Germany) for experiments with freshwater microalgae and cyanobacteria, or alternatively 20 ml of f/2 medium (Sigma) in the case of their marine counterparts. Flasks were initially placed at 22°C under a continuous photon flux density of 60 µmol m^−2^ s^−1^ over the waveband 400–700 nm provided by cool white fluorescent tubes. Cultures were maintained in balanced growth corresponding to mid-log exponential growth by serial transfers of a cell inoculum to fresh medium.

### (b) Experimental design

Most phytoplankton groups have a great phenotypic plasticity for physiological acclimation to changes in their habitat conditions, which is supported by modifications of gene expression [19]. Nevertheless, when these changes exceed the physiological limits, species survival depends exclusively on adaptive evolution, which is in turn driven by the occurrence of mutations that confer resistance [20]. It is difficult to experimentally estimate the optimum selection pressure that ensures enough events of adaptive mutations is difficult as strong selection pressures drastically reduce population size. This constraint can be overcome by performing experiments that include several levels of the selection agent. Accordingly, an experimental procedure was developed [21]—the so-called ratchet protocol—based on the exposure of large populations of single species to short-term intense selection, which was attained by maintaining a strong selection pressure at a temporal scale up to several months. This technique was subsequently improved [22] through modifications in the original design to maximize the occurrence of mutants and their concomitant selection by applying variable selection pressures. This enhancement was achieved by simply using different replicates of each species under the selecting condition, thereby assuring repeatability. The ratchet protocol has been applied in a number of studies to characterize the adaptation of microalgae to extremely hostile habitats [22–27].

Hence, the maximum capacity of phytoplankton to adapt to a warming process can be assessed experimentally through this procedure by analysing the growth of individual species subjected to increasing temperature (as the selecting agent) during many generations. The ratchet protocol permits selection and preservation of the occurrence of both pre-existing and arising mutations that benefit the population and lead to thermal adaptation. Even though this evolutionary approach may be considered an oversimplification of the natural scenario, it still provides a good approximation to the initial stage encountered by an organism in the field when temperature progressively varies.

The procedure followed in this work was aimed at reaching equilibrium between strong selection pressure, by means of ratcheting species to a warmer temperature, and the maintenance of a population size large enough to ensure the occurrence of mutations conferring adaptation. Thus, cultures of individual species were ratcheted only up to a temperature that supported population growth and were exposed to different selection levels. Sixteen independent experiments were conducted (one for each phytoplankton strain). During the early phase, three replicates of control cultures containing growth medium and three replicates of cultures for each temperature value were prepared (see the electronic supplementary material, figure S1). Three initial temperatures were set up at 22°C, 30°C and 35°C. Replicates were grown separately in 5 ml tubes (Sarstedt, Nümbrecht, Germany) inoculated with 3 × 10^5^ cells ml^−1^ of the wild-type population from mid-log exponential growing cultures. This cell concentration was considered large enough to ensure the occurrence of a large final population after applying a temperature rise. In the case of *Symbiodinium* sp. the initial cell density used as inoculum was 10^5^ cells ml^−1^, owing to the lower growth saturation of this species.

All cultures were counted using a particle counter (Beckman Z2, Brea, CA, USA), except for *S. intermedius* cultures, which were counted using a haemocytometer and an inverted microscope (Axiovert 35, Zeiss, Oberkochen, Germany). Cultures were kept under the selecting temperature value for 15–20 days prior to observation. At this stage, cell concentrations were again counted, and comparison between control and experimental cultures was made. If cell concentration in one of the replicates was similar to or higher than that in control tubes (estimated by mean comparisons of 15 countings using Student's *t*-test), it could be assumed that noticeable growth had been achieved by the population under the warmer temperature. The replicate was then ratcheted to the next temperature cycle and subjected to a higher temperature. Replicates that did not reach a cell concentration equivalent to that found in wild-type populations (control cultures) were not transferred (see the electronic supplementary material, figure S1).

In this procedure, each individual tube is considered an independent population. Therefore, if cell density in one of the three replicates belonging to the same initial set was similar to that achieved in control cultures, that particular replicate was ratcheted to the next temperature cycle, regardless of the cell density existing in the other two replicates. This criterion was followed to select different resistance levels that could be attained separately or resistant microalgae likely to occur earlier. In other words, each tube presented a different random chance for particular beneficial mutations, which may arise individually. Both control and ratcheted cultures were again inoculated in this second stage with identical cell concentrations to those used during the first cycle.

A ratchet cycle was concluded when no further cell growth was observed to proceed in a replicate after a period of 100 days. The number of ratchet cycles was then species-dependent as growth was the result of the different adaptation capacity to temperature. The maximum level of resistance of each species was estimated as the highest temperature that allowed the occurrence and growth of a resistant genotype.

Growth rates were calculated before and after the ratchet experiments at the final temperatures of the cycles according to the equation *r* = log_e_(*N*_*t*_/*N*_0_)/*t*, where *t* = 5*d*, and *N*_0_ and *N*_*t*_ are the cell density at the start and at the end of the experiment, respectively. The number of generations during the ratchet experiments was estimated as in [28].

## 3. Results and discussion

### (a) Interpretation of the selection experiments (phenotypic acclimation and genetic adaptation)

Survival of phytoplankton under temperature increase involves a complex combination of phenotypic acclimation, mutation and selection. Although micro-organisms can survive in unfavourable environments as a result of phenotypic acclimation, which is driven by physiological modifications without genetic changes, when the threshold of an environmental factor exceeds the physiological limits, survival depends exclusively on genetic adaptation, supported by the occurrence of mutations that confer resistance and subsequent selection [20]. Whereas the neo-Darwinian view postulating that adaptation to unfavourable environments occurs by selection on new mutations was widely accepted by the 1940s, many biologists felt that adaptation in microbes (including phytoplankton) might take place through a physiological process [29]. Nevertheless, the unifying neo-Darwinian principles have been experimentally confirmed ever since in numerous studies on phytoplankton adaptation [22–27].

The ratchet protocol has been specifically designed as a tool to estimate the maximum capability for adaptation in phytoplankton, which is obtained by genetic adaptation [29]. Single cells are used to inoculate triplicates of a particular strain, thereby ensuring that initial cultures are clonal (containing only one genotype). Since the propagation of beneficial mutations allows survival at increasing temperatures, the potential for adaptation to a temperature rise is experimentally assessed by maintaining populations large enough to maximize the occurrence of those beneficial mutations under strong selection pressure and to favour their enrichment within populations. Although the ratchet procedure is unable to disentangle the relative contributions of physiological acclimation and genetic adaptation, two distinct results can be found by performing the ratchet experiments, which can be interpreted as the independent consequences of two different phenomena: phenotypic acclimation occurring at physiological level without genetic changes, or genetic adaptation owing to the appearance of new mutations that confer resistance, followed by selection of mutant genotypes [20].

In the first case, if resistant cells arose exclusively by physiological acclimation, the number of generations required to grow under temperature increase should be identical in all replicates of a particular strain, because each individual cell has the same chance of developing resistance. In contrast, if resistant cells arose by mutation, the number of generations required to grow under temperature increase should be different among the replicates of each strain. This effect is due to the fact that mutations appear at different times in the replicates, or perhaps mutational events may not even take place. As indicated in table 2, our results show that the number of generations required to proliferate under a temperature rise differed between triplicates of each strain. Although acclimation and mutation can happen simultaneously, the inter-replicate variability observed in all the strains can only be explained if rare spontaneous mutations are involved in adaptation to the progressive warming. Experimental measures of mutation rates in phytoplankton range from 10^−5^ to 10^−7^ mutations per cell per generation [22–27]. Therefore, the high cell density maintained during the experiments presumably assured the appearance of numerous mutants, which propagated through subsequent generations under the strong selection pressure provided by the ratchet cycles.

**Table 2.** Number of generations (*g*) required to grow under increasing temperature during the ratchet experiment cycles.

isolation site	strain	replicate	22 → 30°C	30 → 35°C	35 → 40°C	40 → 45°C
continental water bodies	*Scenedesmus intermedius*	no. 1	15	30	135	—
		no. 2	15	30	135	—
		no. 3	15	30	150	—
	*Dictyosphaerium chlorelloides*	no. 1	15	90	—	
		no. 2	15	120	—	
		no. 3	15	90	—	
	*Microcystis aeruginosa* (*Ma3D*)	no. 1	8	24	—	
		no. 2	8	24	—	
		no. 3	8	16	—	
	*Microcystis aeruginosa* (*Ma6D*)	no. 1	8	24	—	
		no. 2	8	24	—	
		no. 3	8	32	—	
	*Microcystis aeruginosa* (*Ma7D*)	no. 1	15	38	—	
		no. 2	15	45	—	
		no. 3	15	45	—	
open ocean	*Emiliania huxleyi* (*CCMP 371*)	no. 1	—			
		no. 2	—			
		no. 3	—			
	*Emiliania huxleyi* (*CCMP 372*)	no. 1	—			
		no. 2	—			
		no. 3	—			
	*Isochrysis galbana*	no. 1	10	50	—	
		no. 2	10	50	—	
		no. 3	10	40	—	
	*Monochrysis lutheri*	no. 1	—			
		no. 2	—			
		no. 3	—			
coastal waters	*Tetraselmis suecica*	no. 1	15	90	—	
		no. 2	15	90	—	
		no. 3	15	120	—	
	*Phaeodactylum tricornutum*	no. 1	—			
		no. 2	—			
		no. 3	—			
	*Prorocentrum triestinum*	no. 1	25	—		
		no. 2	25	—		
		no. 3	30	—		
	*Nitzschia closterium*	no. 1	20	—		
		no. 2	30	—		
		no. 3	20	—		
	*Navicula* sp.	no. 1	27	—		
		no. 2	34	—		
		no. 3	20	—		
corals	*Symbiodinium* sp. (*CCMP 2429*)	no. 1	65	—		
		no. 2	55	—		
		no. 3	60	—		
	*Symbiodinium* sp. (*CCMP 2433*)	no. 1	60	—		
		no. 2	70	—		
		no. 3	65	—		

Even though each replicate exhibited a different number of generations, triplicates of the same strain invariably reached the same range of temperature tolerance. This repeatability indicates that the ratchet procedure is a good estimator of the maximum capability for adaptation.

Additionally, growth rates of the different strains were measured under the assayed temperatures prior to (ancestral strains) and after (derived strains) the ratchet selection experiments (table 3). If a phytoplankton species was able to survive in an unfavourable temperature only as a result of phenotypic acclimation (physiological non-genetic changes), then the genetically unchanged ancestral genotypes would grow at the same speed after being subjected to a ratchet cycle, and its optimum growth temperature would remain also unmovable. However, growth for genotypes derived from temperature selection obtained after the ratchet experiments showed a very different pattern, in terms of both growth rates and optimum temperatures, than ancestral genotypes prior to the ratchet experiments (table 3). For instance, derived strains of *S. intermedius* were able to grow rapidly at 40°C while ancestral strains were not (table 3). Derived strains of *D. chlorelloides*, *M. aeruginosa*, *I. galbana* and *T. suecica* occurred at 35°C, whereas their respective ancestral strains were unable to grow at that temperature (table 3). A similar response was observed in *P. triestinum*, *N. closterium*, *Navicula* sp. and *Symbiodinium* sp. (table 3). These qualitative differences between ancestral strains prior to the ratchet experiments and derived strains after the ratchet experiments corroborate that adaptation was indeed reached by a genetic change (mutation + selection).

**Table 3.** Growth rates of the different strains under the temperatures assayed prior and after the ratchet experiments (*u*, unable to grow).

species	growth rate					
	ancestral strains (before ratchet experiments)			derived strains (after ratchet experiments)		
	30°C	35°C	40°C	30°C	35°C	40°C
*Scenedesmus intermedius*	0.52	0.46	*u*	0.46	0.50	0.41
*Dictyosphaerium chlorelloides*	0.53	*u*		0.53	0.48	*u*
*Microcystis aeruginosa* (*Ma3D*)	0.28	*u*		0.29	0.22	*u*
*Microcystis aeruginosa* (*Ma6D*)	0.25	*u*		0.27	0.20	*u*
*Microcystis aeruginosa* (*Ma7D*)	0.27	*u*		0.30	0.21	*u*
*Emiliania huxleyi* (*CCMP 371*)	*u*			*u*		
*Emiliania huxleyi* (*CCMP 372*)	*u*			*u*		
*Isochrysis galbana*	0.17	*u*		0.18	0.11	*u*
*Monochrysis lutheri*	*u*			*u*		
*Tetraselmis suecica*	0.48	*u*		0.51	0.42	*u*
*Phaeodactylum tricornutum*	*u*			*u*		
*Prorocentrum triestinum*	*u*			0.16	*u*	
*Nitzschia closterium*	*u*			0.35	*u*	
*Navicula* sp.	*u*			0.21	*u*	
*Symbiodinium* sp. (*CCMP 2429*)	*u*			0.17	*u*	
*Symbiodinium* sp. (*CCMP 2433*)	*u*			0.19	*u*	

In connection with these results, a study aimed at disentangling the effects of physiology, mutation, selection, chance and history in adaptation to temperature increase and eutrophication in marine dinoflagellates has provided evidence of almost no contribution of physiology, chance or history to this process [30]. Also, Gould [31] proposed a theoretical experiment consisting in ‘replaying life's tape’ to unravel the effects of the aforementioned factors on evolutionary change. His theoretical proposal was empirically addressed by a robust experiment in which, instead of ‘replaying life's tape’ sequentially, the same objective was achieved by replicating independent isolates propagated simultaneously [32]. Recently, a similar experiment designed to examine the effect of temperature and eutrophication on toxin production in several strains of *M. aeruginosa* has shown that adaptation occurred through new mutations arising during propagation of cultures under the selecting conditions, which displaced the wild-type ancestral genotypes [33].

### (b) Specific growth responses to temperature

When the usual threshold of an environmental condition changes, the most sensitive organisms are excluded and the most resistant individuals become favoured. This mechanism increases community tolerance and contributes to alter its own structure, exerting a differential selection pressure on community diversity [19].

In addition to this ecosystem response, an intraspecific selection pressure occurs and resistant genotypes are selected. The growth responses obtained here when cells were successively exposed to increased temperatures seem to be coherent with this pattern. By raising the temperature in consecutive cycles, the ratchet technique resulted in a relatively rapid evolution of phytoplankters, although each species showed a particular level of thermal resistance (table 2), which was achieved by genetic adaptation through the appearance of mutants that displayed different growth requirements than their respective parental genotypes (table 3).

Results reveal that, on the grounds of temperature alone, there are clear interspecific differences in phytoplankton survival of a gradual warming process (table 2). Phytoplankton species isolated from continental water bodies characterized by a wide range of temperatures throughout the year (tables 1 and 2) were found to occur at a temperature of 35°C, and even at the highest temperature assayed (as for *S. intermedius*, which resisted up to 40°C). During the first cycle, in which ancestral wild-type strains were ratcheted to 30°C, there were no differences in the number of generations (*g*) between replicates of the same species. The number of generations required to reach the same cell density as in control cultures was 15 in all cases except for *M. aeruginosa* (*Ma3D* and *Ma6D*), which took eight generations (table 2). More differences between replicates of the same species were observed during the second ratchet cycle (from 30°C to 35°C), with *D. chlorelloides* being the organism that needed a higher number of generations to achieve the cell density found in ancestral populations. However, in all cases, *g* rose in relation to that obtained in the first cycle (table 2). In *S. intermedius*, the only species able to efficiently adapt to 40°C after applying a third ratchet cycle, *g* clearly increased in comparison to that needed to adapt to lower temperatures. This organism did not show appreciable growth after ratcheting the temperature to 45°C.

On the other hand, phytoplankton species of open ocean waters exhibited a limited resistance to increased temperatures, and with the exception of *I. galbana*, neither the two strains of *E. huxleyi* nor *M. lutheri* were tolerant of the rise from 22°C to 30°C (table 2). When *I. galbana* was exposed to 35°C, the generations required to achieve the growth of wild-type population notably increased with respect to the first ratchet cycle, being similar among the three replicates (table 2). A third cycle did not lead to growth in this species. The response to warming observed in coral symbionts differed, as the two strains of *Symbiodinium* were able to adapt to 30°C. Nevertheless, it is worth noting that all replicates experienced the highest numbers of generations (approx. 60) to reach cell densities equivalent to those attained by control experiments in relation to all the species that overcame the first cycle (table 2). A second ratchet cycle, increasing the temperature from 30°C to 35°C, did not result in adaptation in this species. Growth of the five phytoplankton species isolated from coastal waters responded differently to warming, and while *T. suecica* was able to resist temperatures up to 35°C, the diatom *P. tricornutum* exclusively proliferated at 22°C (table 2). On the other hand, the other two diatom species *N. closterium* and *Navicula* sp., as well as *P. triestinum*, were tolerant to the rise from 22°C to 30°C. During the first ratchet cycle, *Tetraselmis* exhibited a lower number of generations to grow than the rest of the coastal species, although when this organism was taken from 30°C to 35°C, *g* was found to be six- or eightfold higher (depending on the replicate) than that required during the first cycle.

As indicated above, the design of the ratchet experiments provides additional information, since not only is inter-species or inter-strain variation evaluated, but also the capacity of each replicate to evolve as an independent population. Therefore, the inter-strain variations observed are evidence of the effect of chance on the adaptation process.

The global biogeography of phytoplankton is determined by local environmental factors that select for species based on their optimal growth potential. Among these factors, temperature plays a fundamental role, and in fact the influence of warming on the regulation of phytoplankton dynamics has been reported in aquatic systems including lakes [34] and the open ocean [9,10,17]. Also, the direct influence of temperature on growth rates of microalgae has been broadly evidenced in marine [35] and freshwater species [36]. Consequently, temperature has been always regarded as an effective indicator for phytoplankton distribution in nature. Field observations in continental water bodies and marine systems suggest shifts in phytoplankton occurrence in response to increased water temperature [10–13], although such changes in species succession in the natural habitat are not consistent for all functional groups [18,37], and the general trend described indicates that, in nature, warming favours smaller size classes [16–18]. The majority of studies have been focused on the degree of tolerance to temperature or its effect at ecosystem level rather than on the individual capacity of adaptation. However, both approaches can be combined in order to explain some of the responses already described. Thus, a new model [11] predicts that high temperatures would favour the proliferation of cyanobacteria blooms directly through increased growth rates. The approach proposed by these authors explains the development of the harmful cyanobacterium *Microcystis* during hot summers in eutrophic lakes [11]. The adaptative response of this genus (table 2) is in agreement with the field observations and their modelled tendency, as the three strains of *M. aeruginosa* were able to thrive at temperatures up to 35°C, even though chance affected the adaptation time since both the generations required for optimum growth (table 2) and growth rates (table 3) varied under the new conditions. The fact that this species can genetically adapt to a temperature rise has serious ecological implications in future scenarios, as dense surface blooms of toxic cyanobacteria may lead to mass mortalities of fish and birds, and may represent a serious health threat for cattle, pets and humans [38]. From a classic population genetics point of view, recombination and ploidy must also be taken into account to analyse the speed of adaptive evolution [20]. It is known that haploids respond to selection faster than diploids because non-neutral mutations are quickly expressed. The ploidy of *Microcystis* can therefore be directly related to its ability for mutants to rapidly gain traction in a new environment. Overall, the species with the greatest level of adaptation to warming were haploid populations (table 2).

The rest of the phytoplankton isolated from continental water bodies were also characterized by a great tolerance to high temperatures. In particular, *Scenedesmus*, which even proliferated at 40°C (table 2), possesses a considerable and rapid ability to adjust its cellular physiology, metabolism and growth to relatively large increases in growth temperature [39,40]. Our results indicate that its phenotypic plasticity is based on an elevated capacity for genetic adaptation. Similarly, the cosmopolitan *Dyctiosphaerium* can tolerate a wide range of temperatures, which can be also explained by its rapid genetic adaptation, as already reported in *D. chlorelloides* [41] and corroborated by the ratchet protocol (table 2). This experimental procedure also showed that S*ymbiodinium* occurred up to 30°C, with higher temperatures resulting in a collapse of derived populations (table 2). This finding is in agreement with the fact that photosynthesis in symbiotic dinoflagellates is impaired at temperatures above 30°C and completely ceases at 34–36°C [42]. As warming and photoinhibition are the primary triggers of coral bleaching, many efforts are being devoted to identify the consequences of the heat stress on the symbiotic association between coral and *Symbiodinium*. Previous studies have indicated that the functional response of the symbiosis is indeed temperature-dependent. However, symbiosis breakdown varies between algae of different clades, with some clades being more susceptible to elevated temperatures than others [43,44]. A plausible explanation for distinct heat tolerances may stem from a different thermal sensitivity of the repair of photodamaged photosynthetic machinery among clades. Thus, while severe photoinhibition was observed at temperatures exceeding 32°C in some cells grown at 25–34°C, other more thermally tolerant individuals seemed unaffected [45,46]. In a field transplant study, corals that changed their dominant symbiont type to clade D, a well known thermally tolerant variety of *Symbiodinium*, increased their thermotolerance by 1–1.5°C [47]. In addition, high temperatures have been observed to correlate with the distribution of *Symbiodinium* type in corals, with the symbiont type changing (and possibly conferring thermotolerance) during natural bleaching events. The two strains assayed here could be well integrated into the group of clades more vulnerable to heat stress but with a relatively medium tolerance to a temperature rise in relation to the rest of the species analysed (table 2). The free-living red-tide-forming dinoflagellate *P. triestinum* was also able to adapt to 30°C, coinciding with its habitat preference, as this species is normally found to proliferate during mid-summer in coastal areas characterized by temperatures as high as 30°C [48,49]. Moreover, it appears that dinoflagellates prefer warmer temperatures, which may be a reflection of mixotrophy and the influence of temperature on heterotrophic metabolism or flagellar motility [18]. In contrast, the coccolithophorids considered here displayed a considerable sensitivity to high temperatures, as growth was not measured above 22°C (table 2). Coccolithophores are, on average, most successful (in terms of diversity and proportion of the total phytoplankton community) in warm, oligotrophic, low-latitude waters [50]. It has been suggested that temperature itself plays a direct role in the success of the group, although there is little hard data in support of this contention. *Emiliania huxleyi* thrives even in the relatively cold waters of the north Atlantic south of Iceland, the Patagonian Shelf and the Barents Sea [51]. It seems that, on geological time scales, coccolithophores have adapted to a long-term decrease of atmospheric CO_2_ and cooling ocean temperatures by decreasing their coccolith and cell size. Therefore, if this organism has evolutionarily become adapted to oligotrophic habitats in moderate-temperature waters, it is not surprising that its growth was found to be completely inhibited above 22°C. In fact, cultured *E. huxleyi* has been shown to grow at a temperature range of 10–25°C [52], which is indicative of its preference for warm temperatures and in agreement with our observations. This result contrasts with that found in *I. galbana*, the other haptophyte considered (table 2), which is a non-calcifying species. The distinct temperature tolerance found within this group can be explained on the basis that calcification is strongly influenced by temperature [52,53] and also by taking into account that *I. galbana* occurs in more tropical latitudes than *E. huxleyi* does.

Regarding species isolated from coastal waters, *Tetraselmis* is typical of mid-latitude coastal areas characterized by large annual temperature variations, and, along with *Isochrysis*, has been extensively used in aquaculture worldwide. This circumstance could have favoured the appearance of resistance alleles that have been selected throughout the succession of derived population. Surprisingly, *P. tricornutum* was unable to grow above 22°C, and although this species can cope with higher temperatures, it is also true that its proliferation at 30°C has never been reported. This response contrasts with that exhibited by the other two diatoms, of the genera *Navicula* and *Nitzschia*, which can be considered generalists such as are known to thrive in continental margins ([54] and references therein).

### (c) Implications

The majority of phytoplankters analysed here display a genetic adaptation to warming that seems to be mainly related to the thermal conditions of the natural habitat they have been selected for at a geological time scale. The species that were more flexible and well adapted to the temperature range assayed in this study were those normally encountered in temperate aquatic systems characterized by thermal fluctuations all year round. This finding confirms the accepted view that colder-water communities often lack the genetic redundancy required to withstand an environmental change such as warming. Several studies have highlighted that small organisms are more able to tolerate increased temperature and it has been proposed that global warming will benefit small-sized phytoplankton taxa in aquatic ecosystems [16,17]. An environmental selection towards smaller primary producers would have profound implications for biogeochemical cycles [17,18] and food web structure [13,18]. Nevertheless, our study does not support this notion at an evolutionary level, as the genetic capacity to cope with a temperature rise did not seem to be related to cell size, considering the wide spectrum of cell volumes displayed by the chosen species (table 1). In fact, this trend was also observed intraspecifically, as strains of the same species with different cell volume (e.g. *Microcystis*, *Emiliania* and *Symbiodinium*) evolved in a similar manner under warmer scenarios (tables 1 and 2). These results suggest that the observed shift towards a dominance of small-celled phytoplankton communities [16,17] would have its primary origin in a temperature-driven environmental process, such as nutrient supply or preferential grazing owing to the impact of warming on zooplankton, rather than in a direct thermal effect on phytoplankton metabolism. At present, phytoplankton evolution in a warmer world remains unpredictable, and it is clear that future research must address how the expected temperature rise will alter phytoplankton evolutionary succession. A broad range of work is therefore required to enhance our predictive capabilities. Although the mechanistic technique used in this study constitutes an oversimplification of reality, the evolutionary approach by which the expected temperature rise has been simulated can be considered a novel way to explore the maximum capacity for genetic adaptation to the future thermal scenario in phytoplankton key groups. Our data show that a wide variety of interspecific responses are expected to occur based on the different capabilities of phytoplankters to genetically adapt to a warmer ocean. Such capacity will undoubtedly cause shifts in the composition of the phytoplankton community, as well as replacement of impaired individuals by others that are more resistant; or low-latitude marine species could even colonize higher latitudes as the global sea surface temperature becomes warmer. Although an absolute scenario cannot be envisaged at this point, it is certain that genetics will ultimately determine which species will survive to the environmental forcing.
